# Diel metabolomics analysis of a hot spring chlorophototrophic microbial mat leads to new hypotheses of community member metabolisms

**DOI:** 10.3389/fmicb.2015.00209

**Published:** 2015-04-17

**Authors:** Young-Mo Kim, Shane Nowack, Millie T. Olsen, Eric D. Becraft, Jason M. Wood, Vera Thiel, Isaac Klapper, Michael Kühl, James K. Fredrickson, Donald A. Bryant, David M. Ward, Thomas O. Metz

**Affiliations:** ^1^Biological Sciences Division, Pacific Northwest National LaboratoryRichland, WA, USA; ^2^Department of Land Resources and Environmental Sciences, Montana State UniversityBozeman, MT, USA; ^3^Department of Mathematical Sciences, Montana State UniversityBozeman, MT, USA; ^4^Department of Biochemistry and Molecular Biology, The Pennsylvania State UniversityUniversity Park, PA, USA; ^5^Department of Mathematics, Temple UniversityPhiladelphia, PA, USA; ^6^Marine Biological Section, Department of Biology, University of CopenhagenHelsingør, Denmark; ^7^Plant Functional Biology and Climate Change Cluster, University of Technology SydneyUltimo, NSW, Australia; ^8^Department of Chemistry and Biochemistry, Montana State UniversityBozeman, MT, USA

**Keywords:** gas chromatography-mass spectrometry, metabolomics, microbial mats, polyhydroxyalkanoates, *Roseiflexus*, *Synechococcus*, wax esters

## Abstract

Dynamic environmental factors such as light, nutrients, salt, and temperature continuously affect chlorophototrophic microbial mats, requiring adaptive and acclimative responses to stabilize composition and function. Quantitative metabolomics analysis can provide insights into metabolite dynamics for understanding community response to such changing environmental conditions. In this study, we quantified volatile organic acids, polar metabolites (amino acids, glycolytic and citric acid cycle intermediates, nucleobases, nucleosides, and sugars), wax esters, and polyhydroxyalkanoates, resulting in the identification of 104 metabolites and related molecules in thermal chlorophototrophic microbial mat cores collected over a diel cycle in Mushroom Spring, Yellowstone National Park. A limited number of predominant taxa inhabit this community and their functional potentials have been previously identified through metagenomic and metatranscriptomic analyses and *in situ* metabolisms, and metabolic interactions among these taxa have been hypothesized. Our metabolomics results confirmed the diel cycling of photorespiration (e.g., glycolate) and fermentation (e.g., acetate, propionate, and lactate) products, the carbon storage polymers polyhydroxyalkanoates, and dissolved gasses (e.g., H_2_ and CO_2_) in the waters overlying the mat, which were hypothesized to occur in major mat chlorophototrophic community members. In addition, we have formulated the following new hypotheses: (1) the morning hours are a time of biosynthesis of amino acids, DNA, and RNA; (2) photo-inhibited cells may also produce lactate via fermentation as an alternate metabolism; (3) glycolate and lactate are exchanged among *Synechococcus* and *Roseiflexus* spp.; and (4) fluctuations in many metabolite pools (e.g., wax esters) at different times of day result from species found at different depths within the mat responding to temporal differences in their niches.

## Introduction

Microbial communities inhabiting extreme environments in Yellowstone National Park (YNP) have been investigated for more than half a century (Brock, 1972, 1998). In particular, chlorophototrophic (i.e., chlorophyll-based phototrophs) microbial mat communities present in the effluent channels of Octopus Spring and Mushroom Spring within the Lower Geyser Basin have been intensively studied (Brock, 1978; Ward et al., 2012). As a result of metagenomic (Klatt et al., 2011) and metatranscriptomic (Liu et al., 2011, 2012; Klatt et al., 2013) analyses, an objective and more complete understanding of the major taxa inhabiting the upper 2 mm of the 60–65°C regions of the Mushroom Spring mat, in terms of their contribution to the gene pool and their functional potentials, has emerged (Table 1). Cyanobacteria from the genus *Synechococcus* are the predominant primary producers driving metabolism in these communities via oxygenic photosynthesis (Klatt et al., 2011; Liu et al., 2011). *Synechococcus* spp. fix CO_2_ and synthesize, and possibly excrete, metabolites that are then consumed by (photo)-heterotrophic members of the community, including several Chloroflexi, especially *Roseiflexus* spp. (Table 1), which were formerly thought to be exclusively photoheterotrophs. However, genomics, metagenomics, and metatranscriptomics analyses have revealed that *Roseiflexus* spp. also have the genetic potential to fix CO_2_ (Klatt et al., 2007; Van Der Meer et al., 2010). Collectively, cyanobacteria and *Roseiflexus* spp. account for the majority of the biomass of the upper 0–2 mm portion of the mat community (Table 1), and thus they should have the greatest influence on the metabolites in this portion of the mat. Two additional Chloroflexi, *Chloroflexus* spp. and a novel, apparently phototrophic, Anaerolineae-like taxon, and two aerobic/microaerophilic, anoxygenic photoheterotrophs, Chloracidobacterium thermophilum (Bryant et al., 2007; Garcia Costas et al., 2012) and “*Candidatus* Thermochlorobacter aerophilum” (Liu et al., 2012), also occur in the upper photic layer of the mat. Non-chlorophyllous, heterotrophic bacteria have been detected in the upper mat community, but they are much less abundant (Liu et al., 2011), and are unlikely to strongly influence mat metabolites. Heterotrophs, together with the photoheterotrophic and photomixotrophic community members, can be considered potential consumers of metabolites produced by cyanobacteria and possibly other mat inhabitants.

**Table 1:** **Major members of Mushroom Spring mat photosynthetic layer and their metabolisms**.

**Organism type based on metagenome cluster^a^**	**% of rRNA^b^**	**Mode of primary carbon assimilation in culture^c^**	**Hypothesized mode of primary carbon assimilation in mat^c^**	**Potential carbonaceous substrates consumed based on evidence of ^c^**			**Potential metabolites inferred from genomic/metagenomic analyses**		**Storage polymers inferred from genomic/metagenomic analyses**
				**Transporter genes**	**Pathway genes**	**Use by representative isolates^d^**	**From photorespiration**	**From fermentation**	
*Synechococcus* spp.	27.0	autotrophy	autotrophy	CO_2_, urea, carbohydrates, amino acids	CO/bicarbonate, urea, carbohydrates	CO_2_/bicarbonate	glycolate, glyoxylate, oxalate, glycerate, formate, glycine, serine^e^	lactate, formate, acetate, ethanol	glycogen, cyanophycin
*Roseiflexus* spp.	33.4	heterotrophy	mixotrophy	lactate, fructose, citrate, ornithine, amino acids/peptides, putrescine, spermidine, glucosamine, aminoglycoside, mannitol, trehalose, acetylmuramate, diacetyl chitobiose, melibiose, dimethoxytoluene	CO_2_/bicarbonate, glycolate, lactate, citrate, fructose, amino acids/peptides, ornithine, putrescine, spermidine, glucosamine	fructose, glucose, acetate, pyruvate, lactate, propionate, succinate, malate^f^		lactate, acetate, propionate	glycogen, PHAs, wax esters
*Chloracidobacterium* spp.	10.0	heterotrophy	heterotrophy	oligopeptides, spermidine, putrescine, glutamate, ribose, branched chain amino acids, mannose	mannose, amino acids, glutamate	2−oxo−glutarate, amino acids^h^		lactate, alcohols	glycogen
*Candidatus* Thermochlorobacter spp.	6.0	heterotrophy	heterotrophy	oligopeptides, sugars, oxalate, formate, maltose, acetate, methionine, bicarbonate, glutamate, hydroxymethyl-pyrimidine	amino acids, carbohydrates, formate, acetate, methionine, glutamate, hydroxymethyl-pyrimidine			lactate, acetate, CO_2_	glycogen
*Chloroflexus* spp.	2.5	autotrophy and heterotrophy^g^	mixotrophy	oligopeptides, branched chain amino acids, glutamate, maltose, hydroxymethyl-pyrimidine, ribose, spermidine, putrescine, acetylglucosamine, glycerol−3−phosphate, rhamnose, arabinose	CO_2_, acetate, glycolate, glyoxylate, lactate, glucose, fructose, sucrose, mannose, lactose, galactose, maltose, maltodextrin, trehalose	CO_2_		lactate	glycogen, PHAs, wax esters
Anaerolineae−like	3.8	unknown	unknown						
Heterotroph 1	ND	ND	heterotrophy		glycolate, acetate				
Heterotroph 2	ND	ND	heterotrophy						

a From Klatt et al. (2011).

b From Liu et al. (2012).

c Gene presence is based on annotations of metagenomic clusters and genomes of representative isolates (Klatt et al., 2011, 2013; Liu et al., 2012). Other nutritional requirements, such as those for vitamins, are not considered here.

d Only strains known to be representative of Mushroom Spring populations were considered, including Roseiflexus sp. strains RS1 and RS2 and Chloracidobacterium thermophilum.

e Possible photorespiration products (Bauwe et al., 2010).

f From (Van Der Meer et al., 2010).

g Chloroflexus sp. strain OK-70-fl was reported to grow autotrophically on sulfide and bicarbonate (Madigan and Brock, 1975), and a Chloroflexus sp. isolate from Mushroom Spring can grow on sulfide and carbon dioxide (Thiel et al., unpublished).

h Tank and Bryant, 2015.

ND, not determined.

Studies performed by Konopka (1992) and Nold and Ward (1996) showed that CO_2_-fixing chlorophototrophic community members undergo diel metabolic switching. Recently, metatranscriptomics analyses have provided a comprehensive view of diel transcription patterns in predominant mat taxa (Liu et al., 2011, 2012; Klatt et al., 2013), and have led to new hypotheses about *Synechococcus* spp. and *Roseiflexus* spp. metabolisms. For instance, *Synechococcus* spp. express genes involved in photosynthesis diurnally and have the genetic potential to produce glycogen, which they accumulate during the day (Van Der Meer et al., 2007). Extremely high irradiance during the day leads to O_2_ supersaturation combined with CO_2_ depletion (as indicated by elevated pH), causing production and possible accumulation of toxic levels of glycolate, a common product of photorespiration (Bateson and Ward, 1988). *Synechococcus* spp. also have the genetic potential to conduct fermentation with production of lactate, acetate, ethanol and formate (Bhaya et al., 2007). When photosynthesis declines in the evening, O_2_ uptake by aerobically respiring community members exceeds O_2_ production and the mat becomes anoxic, except within the upper ~150 μm. Fermentation genes, as well as genes involved in N_2_ fixation, are expressed at this time, consistent with measured N_2_ fixation driven by fermentative metabolism at night and by light in the early morning (Steunou et al., 2006, 2008).

Diurnal transcription patterns of the genes involved in CO_2_ fixation suggested that *Roseiflexus* spp. can conduct photomixotrophic metabolism, in which they combine CO_2_ fixation with assimilation of low-molecular weight organic compounds, possibly produced by *Synechococcus* spp. (Klatt et al., 2013). Other transcription patterns suggested that *Roseiflexus* spp. construct and decompose intracellular polymers, including glycogen, polyhydroxyalkanoates (PHAs) and possibly wax esters (genomic and metagenomic analyses show that *Synechococcus* spp. lack the ability to synthesize PHAs (Bhaya et al., 2007; Klatt et al., 2011). Because external reductants such as H_2_ and H_2_S are not present in the oxic mid-day photic layers of the mat, it was further hypothesized that utilization of these intracellular storage polymers may provide reductants and organic intermediates for photomixotrophic CO_2_ incorporation during the day. As suggested by Bauld and Brock (1973), organic compounds produced by CO_2_-fixing community members might be cross-fed to (photo)-heterotrophic or mixotrophic mat community members. Little is known about metabolite exchange in the mat, although it has been shown that acetate, butyrate, ethanol, glycolate, lactate, and propionate are photoassimilated into filamentous community members (Anderson et al., 1987; Bateson and Ward, 1988).

Metabolomics has been successfully applied to characterize the metabolic responses of diverse organisms, both qualitatively and quantitatively, under various growth conditions (Koek et al., 2011). These measurements are increasingly used to study microbial communities (Mosier et al., 2013; Xie et al., 2013). In the current study, a combination of untargeted and targeted metabolomics analyses was performed to quantify five groups of metabolites. Volatile organic acids, polar metabolites, wax esters, and PHAs were measured in the mat, while selected dissolved gasses and inorganic ions were quantified in the overflowing water. Measurements of acetate, propionate, and glycolate in the mat, as well as H_2_, CO_2_, and CH_4_ in the water, were performed to test hypotheses regarding the production of these products during different parts of the diel cycle. Similarly, targeted measurements of wax esters and PHAs were performed to characterize these molecules as intracellular carbon and energy storage polymers that should undergo diel cycling if photomixotrophy occurs as hypothesized in *Roseiflexus* spp. (Klatt et al., 2013). Finally, untargeted metabolomics measurements were performed to identify and quantify polar metabolites extracted from the mat samples (intracellular) and interstitial fluids (extracellular) to identify additional metabolites that are changing during the diel cycle and that may be available for possible metabolic exchange among mat community members, respectively. In addition to evaluating the above hypothesized metabolisms, these data were collectively used to formulate new hypotheses of community metabolisms and metabolite exchange.

## Materials and methods

### Chemicals and materials

All chemicals and reagents were purchased from Sigma-Aldrich (St. Louis, MO) unless otherwise noted. A mixture of fatty acid methyl esters (FAMEs; C8–C28) dissolved in hexane was prepared for use as a retention index standard. PHA polymers were purchased from Sigma-Aldrich or were provided as a gift by Prof. Alexander Steinbüchel at University of Münster, Germany. Deionized and purified water was used to prepare buffer and standard solutions (Milli-Q System Advantage A10, Merck Millipore, Billerica, MA). All solvents and chemicals were obtained in the highest purity available.

### Sample collection

#### Mat samples

**Figure 1:**
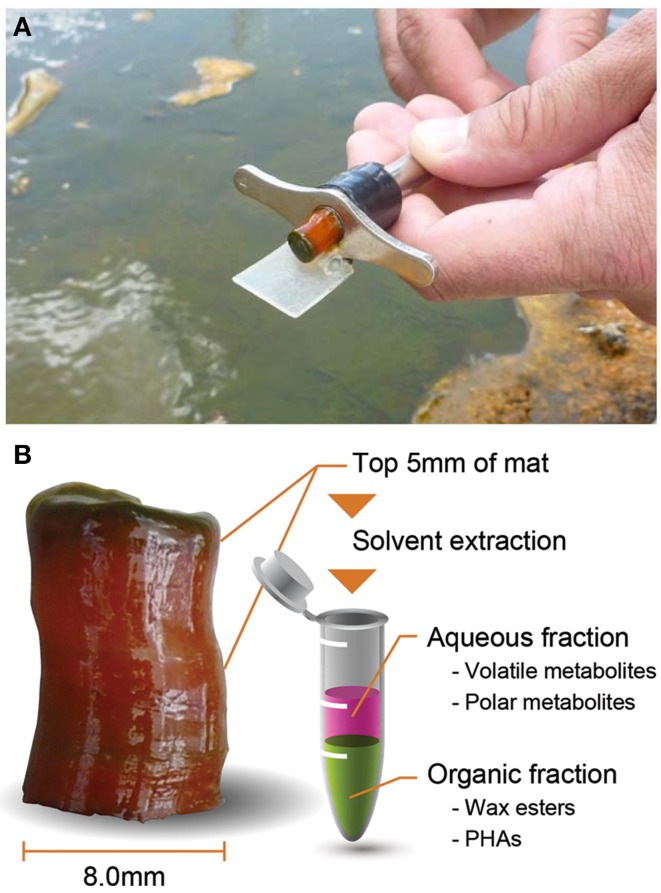
**Core sampling using a cork-borer in Mushroom Spring phototrophic mat (A) and longitudinal view of core sample and brief analytical scheme (B)**.

For whole-mat (i.e., intracellular and extracellular metabolites combined) analyses of volatile organic acids, polar metabolites, wax esters, and PHAs, core samples were collected in from a microbial mat in the effluent channel of Mushroom Spring in the Lower Geyser Basin (YNP, WY) where the temperature of water in the sampling area varied from 58 to 62°C during the diel cycle. A cork-borer with a 8 mm inner diameter was used to collect the same volume of mat sample, and a razor blade was used to separate the top 5 mm of each mat core such that the analyses were focused on the top green phototrophic layer and the red-orange undermat layers in the zone that contain most of the biological activity (Ward et al., 1987) (Figure 1). The samples were transferred to microcentrifuge tubes and immediately frozen in a Dewar containing liquid nitrogen. Mat samples were collected in triplicate at 14 time points between 13:30 h on September 21, 2012 and 11:00 h the following day.

For analyses of extracellular metabolites, mat core samples were collected at 03:00, 09:00, 13:00, and 19:00 h (*n* = 6, each) during the same diel cycle. Once collected, three core samples from each time point were immediately frozen as described above for use as unrinsed controls, while the remaining three samples were transferred to 15 mL Falcon tubes containing 1 mL of spring water that had been filtered through a 0.2-μm filter. Since the 68°C source pool of Mushroom Spring is lined with mat, in order to avoid metabolites that might have diffused from the mat to overflowing water, we used water from the source pool (92°C) of chemically similar Octopus Spring (Papke et al., 2003), which is well upstream of photosynthetic mats (maximum range of 72–74°C). This water did not contain significant levels of any of the organic compounds detected in this study. The re-suspended mat cores were then quickly disrupted onsite by vigorous shaking, and the biomass and rinse water were then immediately separated using a centrifuge (16,025 × *g* for 5 min). The supernatant was transferred to a clean microcentrifuge tube and the rinsed biomass and the rinse water samples were immediately frozen with liquid nitrogen. All samples were stored at −80°C until further processing. This process did not result in release of metabolites identified in analyses of biomass, suggesting that it did not cause leakage of constituents from intact cells.

#### Water samples

Duplicate water samples were collected at 03:00, 07:00, 09:00, 11:00, 13:00, 15:00, 17:00, 19:00, and 23:00 h, during the same diel cycle. The temperature at the collection site was approximately 60°C in the main effluent channel. Channel water was filtered through 0.4 μm HTTP Isopore™ polycarbonate membrane filters, collected in 160-mL serum bottles, and then after several exchanges of the serum-bottle volume, sealed with butyl-acetate stoppers (without head-space).

### Metabolite extraction

A single metabolite extraction protocol was used for the analysis of the various classes of metabolites described herein. Frozen mats were thawed at room temperature and 100 μL each of Nanopure™ water and zirconia-silica beads (0.1 mm size; Biospec Products; Bartlesville, OK) were added, respectively, to the samples and vigorously vortexed for 2 min. This bead-beating process was repeated after the samples were maintained at room temperature for 5 min. A mixture of chloroform/methanol (400 μL; 2:1, v/v) spiked with 20 μg of ^13^C-labeled acetate (Sigma-Aldrich catalog number 282022-250) was added to each disrupted mat sample, and the mixtures were repeatedly vortexed to ensure thorough mixing. The samples were centrifuged at 15,000 × *g* for 5 min at 4°C to separate aqueous and organic layers from precipitated proteins.

For analysis of acetate and propionate, aliquots (50 μL) of the aqueous layer from each extract were transferred to glass vials equipped with glass inserts for direct GC-MS analysis without chemical derivatization. Because acetate and propionate are volatile molecules, all samples were immediately analyzed after extraction.

For untargeted analysis of polar metabolites, aliquots (150 μL) of the remaining aqueous layer from each sample extract were transferred to glass vials and completely dried *in vacuo*. The dried extracts were stored at -20°C until chemical derivatization.

For analysis of wax esters, aliquots (200 μL) of the organic layer from each extract were analyzed directly using GC-MS without chemical derivatization.

For analysis of PHAs, the remaining organic layer from each extract was combined with the corresponding protein pellet, and the combined extract and pellet were completely dried *in vacuo*. The samples were hydrolyzed using a modification of the method reported by Lageveen et al. (1988). Briefly, the dried pellets were dissolved in methanol containing 15% H_2_SO_4_ (v/v) and incubated at 100°C for 15 h. The resulting PHA monomers were extracted with chloroform and analyzed by GC-MS.

### Metabolomics analyses

An Agilent 7890A gas chromatograph coupled with a single quadrupole 5975C mass spectrometer (Agilent Technologies, Inc.) was used for all analyses. Samples were analyzed in duplicate by optimized GC-MS methods, which varied according to the classes of molecular targets as described below.

Acetate and propionate were quantified in a targeted fashion using ^13^C-labeled acetate as an internal standard. Briefly, mixtures of unlabeled acetate and proprionate at different concentrations were combined with constant amounts of ^13^C-labeled acetate in order to construct calibration curves. ^13^C-labeled acetate was then spiked into microbial mat lysates prior to extraction of metabolites, and the measured ratios of unlabeled acetate and propionate to labeled internal standard were used to accurately quantify the target molecules. A polar column (HP-FFAP; 30 m × 0.250 mm × 0.250 μm; Agilent Technologies, Santa Clara) was used. The temperature of the GC inlet was maintained at 200°C, and samples (1 μL) were injected in splitless mode with a helium gas flow rate of 1.0 mL min^−1^. A temperature gradient from 40 to 200°C over 20 min was used, and data were collected over the mass range 20–300 *m/z*. To reduce any carry over arising from the direct injection of the aqueous layers (a mixture of methanol and water) from the metabolite extraction procedure, pure methanol blanks were analyzed between each sample.

For untargeted analysis of polar metabolites, extracted metabolites in the dried aqueous layers were chemically derivatized to trimethylsilyl esters as previously described (Kim et al., 2013). Metabolite extracts were dried *in vacuo* again to remove any residual moisture. To protect carbonyl groups and reduce the number of tautomeric isomers, methoxyamine (20 μL of a 30 mg mL^−1^ stock in pyridine) was added to each sample, followed by incubation at 37°C with shaking for 90 min. To derivatize hydroxyl and amine groups to trimethylsilyated (TMS) forms, *N*-methyl-*N*-(trimethylsilyl)trifluoroacetamide (MSTFA) with 1% trimethylchlorosilane (TMCS) (80 μL) was added to each vial, followed by incubation at 37°C with shaking for 30 min. The samples were allowed to cool to room temperature and were analyzed on the same day. A HP-5MS column (30 m × 0.25 mm × 0.25 μm; Agilent Technologies) was used for untargeted analyses. Samples (1 μL) were injected in splitless mode, and the helium gas flow rate was determined by the Agilent Retention Time Locking function based on analysis of deuterated myristic acid (Agilent Technologies, Santa Clara, CA). The injection port temperature was held at 250°C throughout the analysis. The GC oven was held at 60°C for 1 min after injection, and the temperature was then increased to 325°C by 10°C/min, followed by a 5 min hold at 325°C. Data were collected over the mass range 50–550 *m/z*. A mixture of FAMEs (C8–C28) was analyzed together with the samples for retention index alignment purposes during subsequent data analysis.

For analysis of wax esters, aliquots of the organic layer from the metabolite extracts were directly injected into the GC-MS. For analysis of PHA monomers, the acid-hydrolyzed samples were analyzed. Wax esters and PHA monomers were chromatographically separated using the same HP-5MS column as described above. Samples (1 μL) were injected in splitless mode. The GC oven was held at 60°C for 5 (wax esters) or 10 (PHA monomers) min after injection, and the temperature was then increased to 325°C by 10°C/min, followed by a 1 (PHA monomers) or 5 (wax esters) min hold at 325°C. The helium gas flow rate was 1.0 mL/min and the injection port temperature was held at 250°C throughout the analysis. Data were collected over the mass range 50–600 (PHA monomers) or 50–700 (wax esters) *m/z*.

All GC-MS raw data will be made available via the MetaboLights metabolomics data repository (http://www.ebi.ac.uk/metabolights/) under study identifier MTBLS187.

### Metabolomics data analysis

The relative amounts of acetate and propionate in the mat samples were quantified by isotope dilution mass spectrometry. Standard curves for acetate and propionate were constructed as described above, and the integrated peak areas of acetate, propionate, and ^13^C-acetate in mat samples were determined using the corresponding extracted ion chromatograms (EICs; acetate, *m/z* 60; propionate, *m/z* 74; and ^13^C-acetate, *m/z* 62). The peak areas of endogenous acetate and propionate were divided by that of ^13^C-acetate to obtain ratios of unlabeled/labeled target molecules.

GC-MS raw data files from untargeted analyses of polar metabolites were processed using MetaboliteDetector (Hiller et al., 2009). Retention indices (RI) of detected metabolites were calculated based on the analysis of the FAME standard mixture, followed by their chromatographic alignment across all analyses after deconvolution. Metabolites were then identified by matching GC-MS features (characterized by measured retention indices and mass spectra) to an augmented version of the Agilent Fiehn Metabolomics Retention Time Locked (RTL) Library (Kind et al., 2009), which contains spectra and validated retention indices for over 700 metabolites. All metabolite identifications were manually validated to reduce deconvolution errors during automated data-processing and to eliminate false identifications. The NIST 08 GC-MS library was also used to cross-validate the spectral matching scores obtained using the Agilent library. A heat-map analysis was also carried out after *z*-score transformation of the obtained signal intensities and with K-means clustering (*K* = 5, Distance metric: Euclidean) using DanteR (Taverner et al., 2012).

For wax ester analysis, the 10 most abundant species from >30 detected and quantified in the microbial mat samples were selected based on a previous report (Dobson et al., 1988), and their abundances were determined by the EIC method described above. For PHA analysis, the monomers were also quantified using the EIC method described above. A common representative fragment ion (*m/z* 103) was used for quantifying both 3-hydroxybutyrate and 3-hydroxyvalerate.

### Dissolved gas analysis

Dissolved gasses (CO_2_, H_2_, and CH_4_) were determined using closed head-space GC as described (Inskeep et al., 2005).

### Solar irradiance analysis

The incident downwelling irradiance was logged throughout the field campaign with a LI-1400 light meter equipped with a LI-192 quantum irradiance sensor (LI-COR, Lincoln, NE).

## Results

Triplicate samples were taken from a 60°C region of Mushroom Spring mat at approximately 2-h intervals over a diel cycle (Figure 1). The top 5 mm was removed for solvent extraction and separate analyses of polar and volatile aqueous metabolites, PHAs and wax esters.

### Polar metabolites in the mat

**Figure 2:**
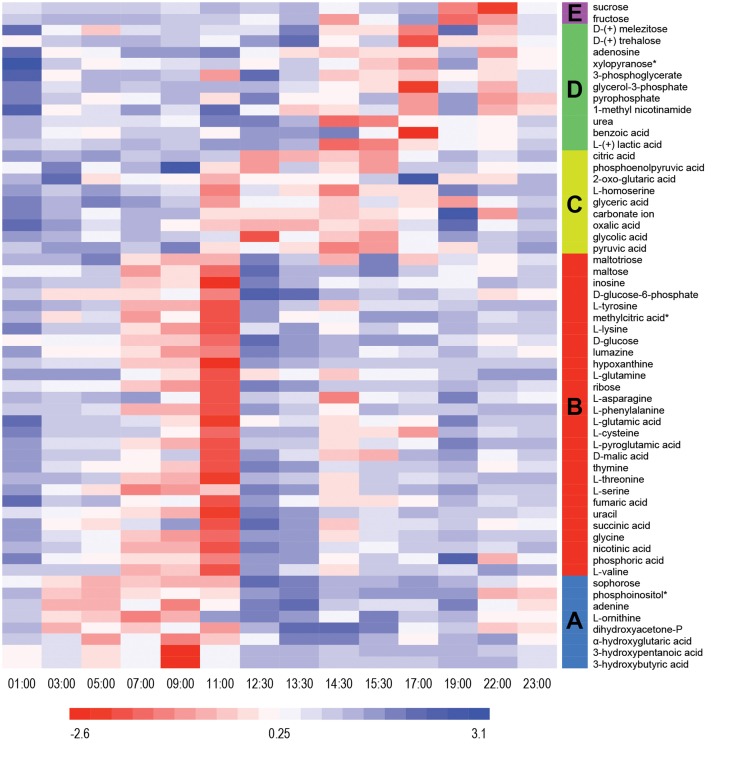
**Heat map view of z-scored polar metabolite abundances over a diel cycle**. A total of 58 metabolites were reproducibly detected (*n* = 3) and confidently identified in the mat samples. The scale bar indicates the z-score transformed average intensity values of metabolites. K-means clustering was performed to categorize the metabolites based on their diel abundance patterns. Cluster A—increase in early morning; Cluster B—increase in late morning; Cluster C—increase in afternoon; Cluster D—increase in late afternoon/early evening; Cluster E—increase at night.

Untargeted metabolomics analyses were performed to identify fluctuations in polar metabolites. This analysis resulted in identification of 58 metabolites that were reproducibly detected in the 42 samples over the diel cycle. The time-course abundance patterns of these 58 metabolites are shown individually in Supplemental Figure S1. K-means clustering was used to categorize these patterns of temporal changes in relative abundances, resulting in five clusters of metabolites (Table 2) that each contained metabolites sharing similar patterns of abundance fluctuation over the diel cycle (Figure 2).

**Table 2:** **List of categorized metabolites showing diel cycling patterns**.

**Cluster**	**Pattern^a^**	**Metabolites**
A (8)	Increase in early morning	adenine
	(03:00–11:00)	dihydroxyacetone phosphate
		3-hydroxybutyric acid
		α-hydroxyglutaric acid
		3-hydroxyvaleric acid
		L-ornithine
		phosphoinositol^*^
		sophorose
B (28)	Increase in late morning	L-asparagine
	(07:00–11:00)	L-cysteine
		fumaric acid
		D-glucose
		D-glucose-6-phsophate
		L-glutamic acid
		L-glutamine
		glycine
		hypoxanthine
		inosine
		lumazine
		L-lysine
		D-malic acid
		maltose
		maltotriose
		methylcitric acid^*^
		nicotinic acid
		L-phenylalanine
		phosphate ion
		L-pyroglutamic acid
		ribose
		L-serine
		succinic acid
		L-threonine
		thymine
		L-tyrosine
		uracil
		L-valine
C (9)	Increase in afternoon	carbonate ion
	(11:00–15:30)	citric acid
		glyceric acid
		glycolic acid
		L-homoserine
		oxalic acid
		2-oxo-glutaric acid
		phosphoenolpyruvic acid
		pyruvic acid
D (11)	Increase in late afternoon/early evening	adenosine
	(14:30–22:00)	benzoic acid
		glycerol-3-phosphate
		L-(+) lactic acid
		D-(+) melezitose
		1-methyl nicotinamide
		3-phosphoglyceric acid
		pyrophosphate
		D-(+) trehalose
		urea
		xylopyranose
E (2)	Increase at night	fructose
	(19:00–22:00)	sucrose

a Clusters are the same as those shown in Figure 2. Numbers in parentheses correspond to the numbers of metabolites comprising the cluster.

* Metabolites identified by the NIST spectral library only.

Metabolites detected in Cluster A accumulated in the predawn and early morning and included 3-hydroxybutyrate and 3-hydroxyvalerate, the monomeric units of PHA. These two compounds showed similar patterns of relative abundance over the diel cycle, as well as to the monomers liberated from acid hydrolysis of intact PHA polymers (see below). Many of the metabolites of Cluster A were lowest in relative abundance in the afternoon and began to increase at 03:00 h, peaking by 09:00 to 11:00 h. Adenine, ornithine (indistinguished from arginine during GC-MS analysis), dihydroxyacetone phosphate, α-hydroxyglutaric acid, sophorose and phosphoinositol showed similar diel profiles.

Cluster B contains metabolites that showed highest abundances in late morning. These included the majority of the amino acids identified in the mat, precursors for the synthesis of nucleic acids, such as hypoxanthine, inosine, phosphoric acid, ribose, thymine, and uracil, as well as intermediates in glycolysis (e.g., glucose and glucose-6-phosphate) and the citric acid cycle (e.g., fumaric, malic, and succinic acids). The abundance profile of maltose, an α-1,4 disaccharide of glucose, paralleled that of glucose, whereas maltotriose, an α-1,4 trisaccharide of glucose, initially increased in abundance, gradually declined and then remained low with minor oscillations throughout the afternoon. A number of metabolites (e.g., asparagine, glycine, malic acid, phenylalanine, succinic acid, threonine, tyrosine, and valine) showed maximal abundances at 11:00 h, followed by an abrupt decrease near mid-day, which was then followed by a secondary maximum around 14:00–15:00 h.

Metabolites assigned to Cluster C showed highest abundance in the early afternoon, a time that correlates to peak photosynthetic activity over the diel cycle (see Discussion). Organic acids such as citric, glyceric, glycolic, oxalic, 2-oxo-glutaric acid (α-ketoglutaric acid), and pyruvic acids were detected in highest abundance during the period of 12:00 to 16:00 h. In contrast to the proteinogenic amino acid serine, the abundance of homoserine was highest from 11:00 to 14:00 h, during which there was an abrupt decrease at mid-day.

Cluster D metabolites accumulated in the late afternoon. Among these, the amounts of lactate and urea dramatically increased from 12:30 to 15:30 h, then gradually decreased until mid-night. In contrast, benzoic acid, glycerol-3-phosphate and trehalose, an α,α-1,1 disaccharide of glucose, showed peak abundance in the early evening (17:00 h).

Only two metabolites, fructose and sucrose, were assigned to cluster E; these accumulated around 19:00 to 22:00 h, decreased at 23:00 h, and then exhibited a relatively low but constant abundance from midnight to noon.

### Volatile fatty acids in the mat

**Figure 3:**
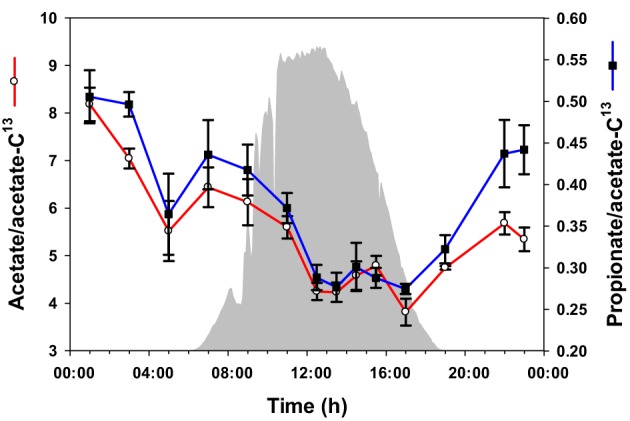
**Volatile metabolites over the diel cycle**. Solar irradiance (solid gray) and acetate and propionate concentrations in unrinsed mat samples are shown. Metabolite values plotted are mean ± standard error (*n* = 3).

To quantify acetate and propionate in the mat accurately, ^13^C-labeled acetate was spiked into samples as an internal standard before metabolite extraction, and the ratios of unlabeled acetate and propionate peak areas to ^13^C-acetate peak area were compared to a calibration curve. Using this quantitative approach, the levels of acetate and propionate were observed to be highest at midnight, followed by a gradual decrease to 17:00 h (Figure 3). Overall, the abundances of acetate and propionate were similar to each other over the diel cycle.

### Carbon storage polymers in the mat

**Figure 4:**
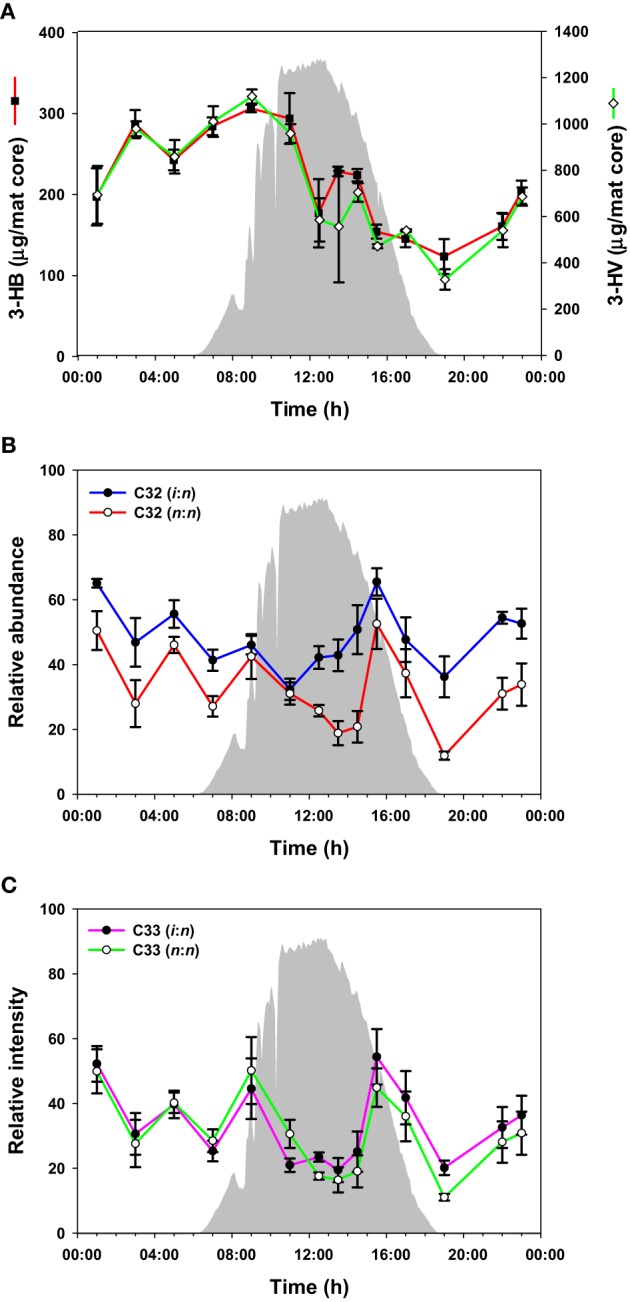
**PHAs and wax esters over the diel cycle**. Solar irradiance (solid gray) and amount of monomers from hydrolysis of PHAs **(A)**; C_32_ wax esters **(B)**; C_33_ wax esters **(C)**. Metabolite values plotted are mean ± standard error (*n* = 3).

PHA was measured over the diel cycle in the form of the major components 3-hydroxybutyric acid (3-HB) and 3-hydroxyvaleric acid (3-HV) (Figure 4A). 3-HV was three times more abundant than 3-HB, with both fluctuating over the diel cycle, although the general trend was for accumulation from 19:00 to 10:00 h followed by a decrease between 10:00 and 19:00 h.

The mat contained a mixture of C_30_-C_35_ *n*,*n* and *i*,*n* wax esters. In total, 42 species were identified (Supplemental Table S1), although we present data for the 10 most abundant species here (representative data shown in Figures 4B,C; all data shown in Supplemental Figure S2). The abundances of these wax esters changed throughout the diel cycle in a complex pattern. In general, the wax ester abundances showed decreases from mid-night to mid-day, except for increases in morning and afternoon, followed by an increase again in the evening. Interestingly, *i,n* forms of C_31_, C_32_, and C_35_ wax esters increased before *n,n* forms.

### Metabolite partitioning in the mat

**Figure 5:**
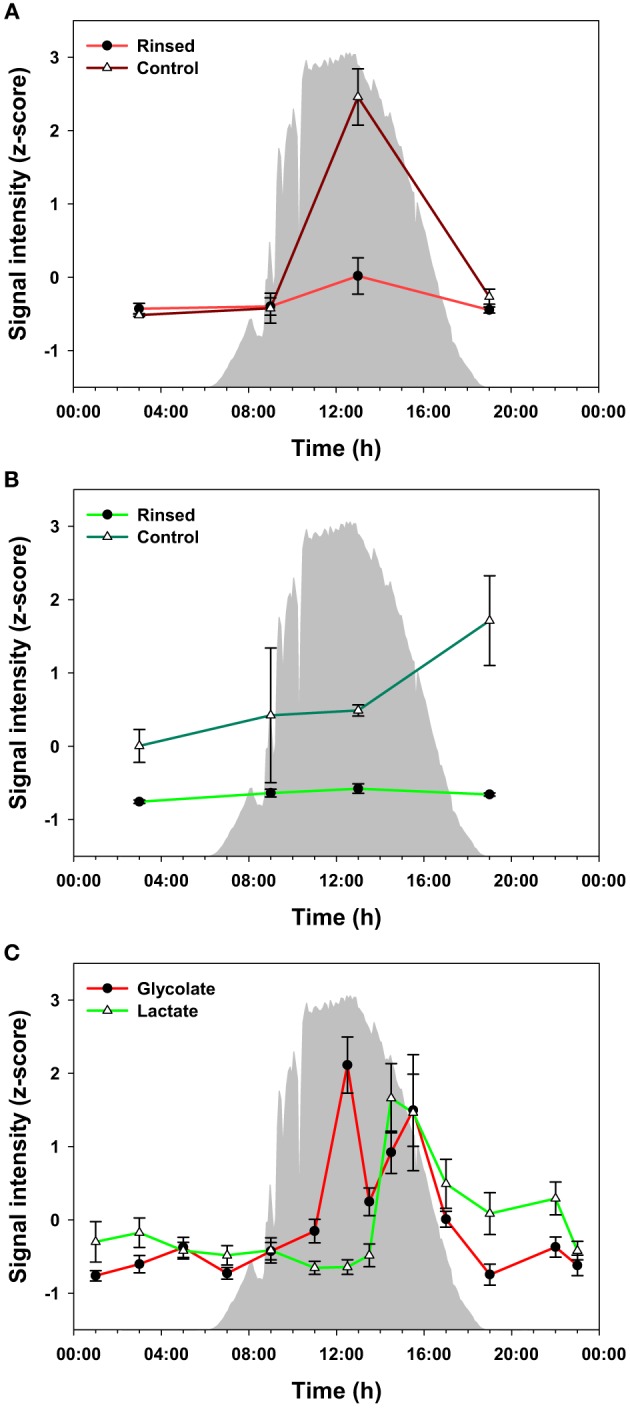
**Measured levels of glycolate (A) and lactate (B) from the rinsed and control (un-rinsed) mats**. The difference between the two conditions is regarded as a portion biologically available by excretion and diffusion in the mats, which can be taken up by other heterotrophic bacteria in the communities. Glycolate and lactate profiles in the unrinsed mat over the full diel cycle **(C)** are shown as a reference. The glycolate and lactate abundances were z-score transformed (i.e., normalized), and the values plotted are mean ± standard error (*n* = 3). Solar irradiance is shown in solid gray.

To evaluate the potential for metabolite exchange among members of the community, we analyzed additional mat core samples that were collected during four time points over the diel cycle and measured metabolites that were excreted or were otherwise extracellular. For this experiment, warm, filtered hot spring water was used to rinse the mat samples on site to avoid release of metabolites due to osmotic shock. Glycolate and lactate were the only metabolites confidently identified in the rinse waters within the detection limits of our instrumentation (data not shown). We compared the levels of these metabolites between the rinsed and control mats (Figures 5A,B) at 4 time points during the diel cycle. The highest level of glycolate in control and rinsed mat samples occurred at 13:00 h. Otherwise, the level of glycolate was relatively the same at 03:00, 09:00, and 19:00 h. In contrast, the abundance of lactate in the rinsed mat samples was equal across the four time points. The levels of lactate in the control mat were much higher than in the rinsed mat and increased from 03:00 to 19:00 h. Figure 5C shows data for glycolate and lactate over the full diel in unrinsed mat samples. The diel trends for glycolate in Figure 5A (control mat) and Figure 5C (unrinsed samples from the full diel sampling) clearly track each other. Similarly, the data for lactate in control mat from the rinsing experiment (Figure 5B) shows a rise in lactate abundance beginning at 13:00 h, which matches the time of the rise in lactate in the unrinsed samples from the full diel sample collection (Figure 5C). However, while the lactate abundance continues to rise to 19:00 h in the control mat from the rinsing experiment, it has begun to decline by 15:30 h in the unrinsed samples from the full diel experiment.

### Gases in the overflowing water

**Figure 6:**
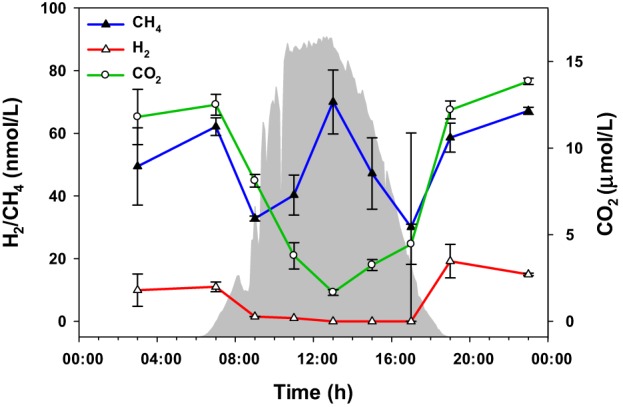
**Gaseous metabolites over the diel cycle**. Solar irradiance (solid gray) and CO_2_, H_2_, and CH_4_ levels in water overflowing the mat are shown. Metabolite values plotted are mean ± standard error (*n* = 3).

The amounts of three gaseous molecules in the water overflowing the 60°C mat—CO_2_, H_2_, and CH_4_—were also measured over the diel cycle (Figure 6). While the levels of hydrogen and carbon dioxide were lower during the day, methane abundance fluctuated, with maxima at 07:00, 12:30, and 23:00 h.

## Discussion

The application of systems biology approaches is expanding from lab-cultured samples to complex environmental communities. In this way, integrated studies are becoming more common for understanding biological systems through the combination of data from metagenomics, metatranscriptomics, metaproteomics, and metametabolomics analyses. Interpreting data from metabolomics analyses of a complex microbial community is challenging because many taxa may contribute to metabolite pools and because they may do so at different times during a diel cycle. Furthermore, metabolite concentrations represent pools that are influenced by production and consumption, as well as by diffusion, and all three factors are closely coupled in aquatic microbial mats. Thus, metabolite fluctuations with time likely represent periods of net production/accumulation or consumption/diffusion. Nevertheless, the data obtained in this study supported existing hypothesized metabolisms of major taxa in the mat and led to new hypotheses based on novel observations, as discussed below.

### Integration of metabolomics and gene expression data: support of existing hypotheses on *synechococcus* spp. and *roseiflexus* spp. metabolisms within the mat community

In this section, we interpret certain metabolomics results in the context of hypotheses generated from previous diel metatranscriptomics studies (Liu et al., 2011, 2012; Klatt et al., 2013). Although the metatranscriptomics results are from a different year (September 2009), the long-term stability of the mat community, its composition and structure (Ramsing et al., 2000; Ferris et al., 2003; Ward et al., 2006; Becraft et al., 2011; Melendrez et al., 2011), processes conducted during diel cycles by phototrophic community members based on O_2_ concentration profiles, and expression of *Synechococcus* photosynthesis and N_2_ fixation genes (Ramsing et al., 2000; Ward et al., 2006; Steunou et al., 2008; Jensen et al., 2011; Liu et al., 2011, 2012) as measured between 1996 and the present, make comparisons of data collected at comparable temperature sites and times of the year valid. Indeed, comparison of solar irradiance and glycolate levels over a diel cycle in mat samples collected in 2011 showed very similar abundance profiles as the data presented here (Supplemental Figure S3).

### *Synechococcus* spp.

Based on diel changes in glycogen (Van Der Meer et al., 2007) and metatranscriptomics analyses (Liu et al., 2012), we hypothesized that *Synechococcus* spp. shift from daytime photosynthesis and the production of glycogen to nighttime glycogen fermentation (Van Der Meer et al., 2007). Consistent with this hypothesis, fermentation products that mat *Synechococcus* populations have the genetic potential to produce (e.g., acetate and lactate) accumulated during the afternoon and night (Figures 3, 5C).

Mid-day extremes of light and O_2_ concentration, as well as CO_2_ depletion (suggested by a rise in pH, which shifts the carbonate equilibrium) have been shown to lead to photorespiratory production of glycolate (Bateson and Ward, 1988). Thus, we hypothesized that *Synechococcus* spp. in the mat experience photorespiration during periods of high light irradiance. Supporting this hypothesis, CO_2_ in the water flowing over the mat decreased during the day (Figure 6), and glycolate accumulated between ~12:00 and ~16:00 h (Figure 5C). Production of glycolate at peak solar irradiance correlated with the expression of *Synechococcus* spp. genes encoding photosynthesis machinery (Liu et al., 2012).

Additionally, nighttime and early morning N_2_ fixation by *Synechococcus* has been demonstrated (Steunou et al., 2006, 2008), and because mat *Synechococcus* lack an uptake hydrogenase, we hypothesized that H_2_ accumulation should temporally follow N_2_ fixation. Diel patterns of H_2_ concentration in the water above the mat (Figure 6) are consistent with this prediction.

### *Roseiflexus* spp.

Noting the diel cycling of transcript abundances encoding enzymes associated with the 3-hydroxypropionate pathway and the production and consumption of polymers known to be produced by *Roseiflexus* spp., Klatt et al. (2013) hypothesized that *Roseiflexus* spp. shift from a photomixotrophic metabolism leading to glycogen synthesis during the day to nighttime fermentation of glycogen, coupled with nighttime synthesis of PHA and/or wax esters, whose breakdown during the day could in turn provide the necessary metabolites for photomixotrophy. Consistent with this hypothesis, diel glycogen cycling was previously demonstrated by Van Der Meer et al. (2007). Also consistent with the hypothesis, levels of CO_2_ in the water overflowing the mat and of intracellular fermentation products known to be used by *Roseiflexus* [e.g., acetate, propionate, and lactate; based on genomic (Van Der Meer et al., 2010; Bryant et al., 2012) and metagenomic (Klatt et al., 2011) analyses and on laboratory growth experiments (Hanada et al., 2002)] are lower during the day.

In addition, PHAs, measured by their constituent monomers (e.g., 3-HB and 3-HV) after acid hydrolysis of the polymers, were relatively higher at night and in the early morning, followed by a decrease during the day (Figure 4A). The accumulation of 3-HB and 3-HV as free monomers in the morning (Figure 2, Supplemental Figure S1), together with methyl-citrate, an intermediate in the oxidation of propionate (which could be derived from 3-HV), provides evidence that PHAs are being degraded in the early morning. These observations are consistent with previous metatranscriptomics data on expression of *Roseiflexus* spp. PHA biosynthesis genes, and our previous hypothesis that these molecules might be used for mixotrophic metabolism by filamentous anoxygenic phototrophic bacteria (Klatt et al., 2013).

Wax esters generally cycled in a manner consistent with the expression patterns of *Roseiflexus* genes associated with their production and degradation, supporting their hypothesized involvement in photomixotrophy. However, these compounds fluctuated in a complex manner, possibly reflecting differences due to the timing of metabolisms of different *Roseiflexus* species (see below).

#### Novel observations leading to new hypotheses

In this section, we highlight novel observations of metabolism in the Mushroom Spring microbial mat community with respect to metabolites identified or measured for the first time, as well as to the time of day at which certain metabolites showed peaks in accumulation. These observations were then used as the basis upon which new hypotheses have been formulated.

#### Detection and accumulation of previously unreported metabolites

The accumulation of CH_4_ in the mat at mid-day was unexpected (Figure 6), since methanogenesis is an anaerobic process that should only occur in the anoxic nighttime mat (Ward, 1978; Sandbeck and Ward, 1981). However, genomic and metagenomic analyses indicate that *Synechococcus* spp. have the potential to metabolize phosphonate (Gomez-Garcia et al., 2011), which can also lead to methane production. We therefore hypothesize that the mid-day peak in methane concentration is a result of *Synechococcus* spp. metabolism of phosphonates.

Metabolites in cluster B accumulated specifically in the morning and in general reached their highest levels at 11:00 h. The metabolites present in this cluster (most amino acids, hypoxanthine, inosine, phosphoric acid, ribose, thymine, and uracil) imply that amino and nucleic acid biosynthesis occur maximally during the early morning period. Interestingly, all of these nitrogen-rich compounds reached peak levels shortly after the maximal period of N_2_ fixation by *Synechococcus* spp., which occurred between 06:00 and 10:00 h in the morning (Steunou et al., 2008). This period also corresponded to the time when total mRNA levels increased sharply in members of the major phototrophic taxa that occur in the mats Liu et al., 2011, 2012; Klatt et al., 2013). While not unexpected, these collective observations lead to the hypothesis that the morning hours represent a time when RNA, DNA, and protein biosynthesis rates are maximal for major taxa in the mat.

At midday (11:00 to 12:00 h) there is an abrupt decline in all metabolites of cluster B, when metabolites of cluster C, including glycolate, oxalate, carbonate, citrate, and phosphoenolpyruvate, accumulated (Figure 2). The accumulation of glycolate (as discussed above), glycerate, and oxalate is likely due to photorespiration by *Synechococcus* spp. (Bateson and Ward, 1988; Bauwe et al., 2010). Interestingly, the abundance of carbonate ion also increased at this time, consistent with extreme CO_2_ consumption and elevated pH during peak periods of photosynthesis shifting the equilibrium of dissolved inorganic carbon (Revsbech and Ward, 1984; Jensen et al., 2011). Also of interest is the observation that peak production of glycolate coincides with the abrupt decrease in levels of certain metabolites (asparagine, glycine, malic acid, phenylalanine, succinic acid, threonine, tyrosine, and valine) in cluster B, suggesting a decrease in activity in these metabolic pathways possibly due to photoinhibition. At the same time as the abrupt decrease in abundances of cluster B metabolites and just after the peak in glycolate abundance (~12:00 h), the levels of lactate in the mat begin to increase, with maximal abundance at ~15:00 h and correlating with a second peak in glycolate abundance (Figure 5C). We hypothesize that *Synechococcus* spp. may be a source of the peak in lactate abundance at this time via fermentation either as an alternative metabolism for photoinhibited cells closest to the mat surface, or because cells deeper in the mat experience a shorter period of peak solar irradiance (Becraft et al., this issue; Olsen et al., this issue), or both.

#### Metabolite exchange

Metabolic interactions among community members are key features stabilizing the composition and function of microbial communities. In a chlorophototrophic microbial community, organic compounds produced and excreted by CO_2_-fixing taxa could be used as nutrients by (photo)-heterotrophic or mixotrophic mat community members. Indeed, diurnal transcription patterns of the genes involved in CO_2_ fixation have suggested that *Roseiflexus* spp. in the Mushroom Spring mat community can conduct photomixotrophic metabolism, presumably using organic compounds produced and excreted by other community members. In this section, we discuss the potential for metabolic exchange between *Synechococcus* and *Roseiflexus* spp.

Two metabolites, the photorespiration product glycolate and the fermentation product lactate, were identified in the extracellular fractions of the rinsing experiment and were therefore available as nutrients for members of the mat community. Glycolate was most abundant in the mat during the early afternoon (Figures 2, 5C, and Supplemental Figure S1). At 13:00 h, the amount of glycolate associated with mat biomass was much lower (25–30%) in the rinsed compared to the unrinsed control samples, suggesting that glycolate is excreted into the intracellular milieu (Figure 5A). At other time points examined, the amounts of glycolate were similar in rinsed or unrinsed samples, suggesting a balanced consumption and production or that photorespiration is less active at lower irradiance levels. Although, Klatt et al. (2013) did not observe significant changes in transcription patterns in *Roseiflexus* spp. during the same time period as the peak in mat glycolate abundance, these organisms are still the most likely consumers of glycolate because glyoxylate derived from glycolate by oxidation can readily be assimilated by the 3-hydroxypropionate bi-cycle (Klatt et al., 2007). In contrast, a very sharp and large increase (~60-fold above the minimum) in transcript abundance for lactate permease in *Roseiflexus* sp. at approximately 18:00 h has been observed (Bryant et al., unpublished data), just after the afternoon increase in lactate abundance in unrinsed vs. rinsed mat samples in our experiment (Figure 5B). This observation suggests that *Roseiflexus* sp. might utilize a significant proportion of the lactate produced. Indeed, lactate levels declined in the early evening hours after the spike in lactate permease transcripts occurred. As with glycolate, the lower levels of lactate during the night may indicate an efficient balance between production and consumption. Based on these observations, we hypothesize that glycolate, and possibly lactate (as discussed above), are mostly produced and excreted by the cyanobacteria (i.e., *Synechococcus* spp.) during the early afternoon and are available to other mat inhabitants, particularly *Roseiflexus* spp., as a carbon and energy source.

It is interesting that only glycolate and lactate were identified in the rinse water. We have considered several possible explanations for this observation. It is possible that other extracellular metabolites (e.g., volatile fatty acids, ethanol) may have been lost during the *in vacuo* drying of the rinse water samples, as previous analyses have shown that these compounds accumulate in the aqueous fraction during dark, anaerobic incubation of mat samples (Anderson et al., 1987). Alternatively, our sampling of extracellular metabolites, which occurred at 03:00, 09:00, 13:00, and 19:00 h, may not have occurred during the peak times of metabolite excretion. A third possibility is that certain metabolites are rapidly scavenged from the extracellular milieu as soon as they are excreted. The last possibility is that there were no other metabolites that were excreted.

#### Depth- or temporally-resolved metabolisms

As mentioned above, a complex pattern of wax ester abundances was observed, with peak abundances in the predawn, morning, and afternoon periods, and differential timing of *i,n-* and *n,n-*forms of the same wax esters. Such complexity might arise because of contributions from multiple taxa capable of wax ester synthesis with different diel timing. As shown in Table 3, although *Roseiflexus* wax esters are a better match to wax esters found in the mat, *Chloroflexus* also makes *n,n* forms of C_31_, C_32_, and C_35_ wax esters, and the different abundances of these forms might relate to differential timing of wax ester synthesis in members of these two genera. Such could also be the case for different *Roseiflexus* species. Zeng et al. (1992) showed that the ratio of *i,n*- to *n,n*-forms of C_31−35_ wax esters increased nearly 5-fold in mat layers 4–5 mm below the surface of the highly similar Octopus Spring mat, raising the question of whether different species of *Roseiflexus*, with different vertical distributions, experience different light regimes and have different timing of wax ester synthesis and degradation. Taxon-related and/or depth-related differences in metabolisms may be generally important, because similar small-scale fluctuations were observed in PHA, glycolate, and fermentation products. Furthermore, a number of metabolites (e.g., asparagine, glycine, malic acid, phenylalanine, succinic acid, threonine, tyrosine, and valine) showed maxima in abundances at 11:00 h, followed by an abrupt decrease near mid-day, which was then followed by a secondary maximum around 14:00–15:00 h. Metabolomics analyses were conducted on the top 5 mm region of the mat, whereas the transcription results of Klatt et al. (2013) were from the top 2 mm region. Different taxa (and/or different species within these taxa) inhabit different vertical regions of the mat (Ramsing et al., 2000; Becraft et al., 2011), and we hypothesize that they exhibit maximal metabolic rates for specific processes at different times during the diel cycle. Indeed, evidence that *Synechococcus* species with different depth distributions (Becraft et al., this issue), light adaptations (Nowack et al., this issue) and gene expression timing (Olsen et al., this issue), strongly supports this hypothesis.

**Table 3:** **Wax ester content of mat and *Chloroflexi* and timing of abundance changes in the mat**.

**Wax ester**	***Roseiflexus*^a^**	***Chloroflexus*^a^**	**mat^a^**	**Evening rise start**	**Night peak**	**Morning peak**	**Afternoon rise start**	**Afternoon peak**
C31 *n,n*	+		++	19:00	01:00	05:00	14:30	15:30
C31 *i,n*	+		+	17:00	22:00	03:00	11:30	14:30
C32 *n,n*	++	+	+++	19:00	01:00	05:00, 09:00	14:30	15:30
C32 *i,n*	+++		++	19:00	01:00	05:00, 09:00	12:30	15:30
C33 *n,n*	+	+	+++	19:00	01:00	05:00, 09:00	14:30	15:30
C33 *i,n*	+		++	19:00	01:00	05:00, 09:00	14:30	15:30
C34 *n,n*	+++	++	++	19:00	01:00	05:00, 09:00	13:30	15:30
C34 *i,n*	+++		+	19:00	01:00	05:00, 09:00	13:30	15:30
C35 *n,n*	+	++	+	19:00	01:00	05:00, 09:00	15:00	17:00
C35 *i,n*	+		+	19:00	01:00	05:00, 09:00	13:00	17:00
C36 *n,n*		++						
C37 *n,n*		+						

a Relative abundances of wax esters in Roseiflexus spp., Chloroflexus spp., and the Mushroom Spring microbial mat are indicated by “+” for low abundance, “++” for moderate abundance, and “+++” for high abundance. Data for Roseiflexus spp. and Chloroflexus spp. are from Van Der Meer et al. (2010).

### Conflict of interest statement

The Guest Associate Editor, William P. Inskeep, declares that although he has promoted collaboration in this research topic, he is not directly involved with the research reported in this paper, and has no relationship with the independent reviewers who provided comments to the manuscript. He confirms that the review process was handled objectively and that no conflict of interest exists. The authors declare that the research was conducted in the absence of any commercial or financial relationships that could be construed as a potential conflict of interest.
