# Supplementary material for: Diel metabolomics analysis of a hot spring chlorophototrophic microbial mat leads to new hypotheses of community member metabolisms
Source: Front Microbiol. 2015 Apr 17;6:209. doi: 10.3389/fmicb.2015.00209 (PMC4400912; doi:10.3389/fmicb.2015.00209)
Supplement: Supplementary file 1 [file DataSheet1.DOCX]

**Supplemental Table S1. Wax esters identified in the Mushroom Spring microbial mat.**

| **Total chain length** | **Molecular ion**  **(*m/z*)** | **Detected isomers^1^** | **Number of isomers** |
| --- | --- | --- | --- |
| C28 | 424 | *n:n, n:i* | 2 |
| C29 | 438 | *n:n, n:i* | 3 |
| C30 | 452 | *n:n, n:i* | 4 |
| C31 | 466 | *n:n, n:i, i:i* | 5 |
| C32 | 480 | *n:n, n:i, i:i* | 5 |
| C33 | 494 | *n:n, n:i, i:i* | 6 |
| C34 | 508 | *n:n, n:i, i:i* | 6 |
| C35 | 522 | *n:n, n:i, i:i* | 6 |
| C36 | 536 | *n:n, n:i, i:i* | 5 |
| ^1^Structural identification is based on the previous report by Dobson et al (1988). Several structural isomers were detected for each *m/z* and represent different combinations of fatty acids and fatty alcohols. | | | |

**Supplemental Figure legends**

**Supplemental Figure S1.** Individual diel abundance patterns of metabolites in the Mushroom Spring microbial mat comprising the major clusters identified by K-means clustering. *metabolites identified by the NIST spectral library only. The values plotted are mean z-score transformed metabolite peak areas with standard errors (n = 3).

**Supplemental Figure S2.** Diel abundance patterns of the 10 most abundant wax ester species from >30 detected and quantified in the Mushroom Spring microbial mat samples. The values plotted are mean z-score transformed metabolite peak areas with standard errors (n = 3).

**Supplemental Figure S3.** Incident solar irradiance (A) and glycolate abundance (B) profiles from the chlorophototrophic microbial mat of Mushroom Spring, Yellowstone National Park. Collection sites in 12-13 September 2009, 14-15 September 2011, and 21-11 September 2012 were defined by temperature (60°C) rather than location, but were within 2-3 m of each other in the same effluent channel. Similar glycolate accumulation at mid-day was observed for 2009 (data not shown).

**Supplemental Figure S1.**

**Supplemental Figure S2.**

**Supplemental Figure S3.**
